# Towards a patient journey perspective on causes of unplanned readmissions using a classification framework: results of a systematic review with narrative synthesis

**DOI:** 10.1186/s12874-019-0822-9

**Published:** 2019-10-04

**Authors:** R. G. Singotani, F. Karapinar, C. Brouwers, C. Wagner, M. C. de Bruijne

**Affiliations:** 1 Amsterdam UMC, Vrije Universiteit Amsterdam, Department of Public and Occupational Health, Amsterdam Public Health research institute, Van der Boechorststraat 7, NL-1081 BT Amsterdam, The Netherlands; 2 Department of clinical pharmacy, Onze Lieve Vrouwe Gasthuis (OLVG), location West, Jan Tooropstraat 164, 1061 AE Amsterdam, The Netherlands; 3 Netherlands institute for Health Services research, Otterstraat 118-124, 3513 CR Utrecht, The Netherlands

**Keywords:** Hospital readmission, Avoidability, Preventability

## Abstract

**Background:**

Several literature reviews have been published focusing on the prevalence and/or preventability of hospital readmissions. To our knowledge, none focused on the different causes which have been used to evaluate the preventability of readmissions. Insight into the range of causes is crucial to understand the complex nature of readmissions.

With this review we aim to: 1) evaluate the range of causes of unplanned readmissions in a patient journey, and 2) present a cause classification framework that can support future readmission studies.

**Methods:**

A literature search was conducted in PUBMED and EMBASE using “readmission” and “avoidability” or “preventability” as key terms. Studies that specified causes of unplanned readmissions were included. The causes were classified into eight preliminary root causes: Technical, Organization (integrated care), Organization (hospital department level), Human (care provider), Human (informal caregiver), Patient (self-management), Patient (disease), and Other. The root causes were based on expert opinions and the root cause analysis tool of PRISMA (Prevention and Recovery Information System for Monitoring and Analysis). The range of different causes were analyzed using Microsoft Excel.

**Results:**

Forty-five studies that reported 381 causes of readmissions were included. All studies reported causes related to organization of care at the hospital department level. These causes were often reported as preventable. Twenty-two studies included causes related to patient’s self-management and 19 studies reported causes related to patient’s disease. Studies differed in which causes were seen as preventable or unpreventable. None reported causes related to technical failures and causes due to integrated care issues were reported in 18 studies.

**Conclusions:**

This review showed that causes for readmissions were mainly evaluated from a hospital perspective. However, causes beyond the scope of the hospital can also play a major role in unplanned readmissions. Opinions regarding preventability seem to depend on contextual factors of the readmission. This study presents a cause classification framework that could help future readmission studies to gain insight into a broad range of causes for readmissions in a patient journey.

In conclusion, we aimed to: 1) evaluate the range of causes for unplanned readmissions, and 2) present a cause classification framework for causes related to readmissions.

**Electronic supplementary material:**

The online version of this article (10.1186/s12874-019-0822-9) contains supplementary material, which is available to authorized users.

## Background

The definition of unplanned readmissions varies among countries, but it is most often defined as an urgent readmission within 30 days from a previous admission [1–3]. In many cases, unplanned readmissions result in potential health risks for patients, an increased workload for caregivers and excessive healthcare expenditure [4]. Reducing readmission rates is therefore considered to be of great importance. Many countries use readmission rates as a quality indicator of hospital care. However, previous research shows that unplanned readmissions are not only caused by suboptimal care provided by the hospital [5, 6]. In fact, unplanned readmissions that are considered to be potentially preventable due to suboptimal hospital care (~ 20–25% of the unplanned readmissions) often also have other underlying causes which are not related to hospital care [6, 7]. This suggests that hospitals are being held accountable for unplanned readmissions, while they may not always be able to prevent them [8].

In the previous decades, studies have been performed on the prevalence and preventability of readmissions. Existing assessment tools with predefined causes (such as State Action on Avoidable Rehospitalizations (STAAR), Root-Cause Analysis Tool (PRISMA: Prevention and Recovery Information System for Monitoring and Analysis) [9] and Research Electronic Database Capture (REDcap tool) are often applied or adapted in readmission studies. The variation in different sets of causes used makes comparison between studies difficult. Comparison between studies is crucial to fully understand the complex nature of readmissions.

To date, it is unclear which causes are considered in unplanned readmission studies. In particular, the causes which are not related to hospital care have not been given much emphasis so far. Hence, this systematic review focuses on evaluating the different causes that have been taken into consideration in studies on unplanned readmissions using patient record review. With this review we aim to: [1] evaluate the range of causes of unplanned readmissions in a patient journey, and [2] present a cause classification framework that can support future readmission studies.

## Methods

In 2017, we conducted a systematic review on (the implications of) the different methods used to assess the preventability of unplanned readmissions by use of patient record review [10]. Previous studies demonstrated that health administrative databases often lack extensive information (e.g. functional status and social support) [11]. Therefore, we focused on studies which used patient record review to assess preventability of unplanned readmissions. The current systematic review provides an in-depth insight on the causes that have been used by the studies in the previous review.

### Literature search

The literature search was applied in Web of Science, Scopus, Pubmed and Embase in December 2016, using “readmission and avoidability or preventability” as key terms (Additional file 1). A medical information specialist was consulted for the search. All citations were imported into Endnote × 7.3.1TM.

### Study selection

The 77 studies which were included in the previous review [10] were checked for eligibility for this review by two researchers (CB and RS) (see Additional file 2). Studies were included if they fulfilled to the following criteria: original data, primary focus on preventable readmissions, clear description of preventable readmissions and potential causes of preventable readmissions (the cause classification) (see Additional file 3). We defined a cause classification as the description of at least three causes or synonyms for causes (e.g. (contributing) factors and reasons). Studies that described the causes of preventable readmissions (≥3) were further reviewed. Studies that made no distinction in readmissions and (primary) admissions, or preventable readmissions and non-preventable readmissions, were excluded from further analysis. In case of disagreement, a senior researcher was consulted to reach a final selection of studies.

### Critical appraisal of individual studies

We applied a validated approach to evaluate and compare the studies. This approach was developed by the Cochrane Consumers and Communication Review Group with the aim to perform a structured and transparent analysis for systematic reviews that rely primarily on the use of words and text to summarize and explain the findings of the synthesis. The approach can be described as ‘narrative’ analysis or synthesis [12]. We used this approach because of the large heterogeneity in study designs, and because other quality appraisal approaches and tools were less appropriate to apply. Prior to the narrative analysis a data-charting form was jointly developed by the authors to determine which variables to extract (including defining these variables). Subsequently, the authors independently charted the data, discussed the results and continuously updated the data-charting form in an iterative process. The data-charting form included variables such as: study design characteristics, sources of information to assess preventability, definition of preventability and percentage of preventable readmissions. After extracting the data the authors critically evaluated the applicability of the data and tabulated the results in order to identify patterns across the included studies.

### Data collection and classification

#### Preventability

Two researchers (RS and CB) collected all the causes described by the studies. Each cause was classified as preventable or unpreventable. In our previous review, we found that the definition of preventability varied widely among studies. Many studies did not provide a clear definition, but referred to a cause classification with causes that were pre-defined as preventable [10]. For instance, Williams et al. defined potential areas to avoid readmissions; ‘It was noted that readmission could have been avoided if more effective action had been taken in one or more of five areas: preparation for and timing of discharge, attention to the needs of the carer, timely and adequate information to the general practitioner and subsequent action by the general practitioner, sufficient and prompt nursing and social services support, and management of medication’ [13]. Other studies such as Ryan et al. provided a broad definition of preventability; ‘Providers were given no specific guidelines for deciding whether a readmission was preventable. This allowed use of their different backgrounds in choosing which elements of the clinical record to focus on’ [14].

In this study, the researchers coded whether a cause was preventable as stated by the study authors. When the study was unclear regarding the preventability of a cause, the researchers classified the cause as neutral. Causes that studies considered both preventable and unpreventable, were coded as both.

### Root cause

The root causes were based on the PRISMA classification scheme of Schaaf & Habraken [9]. PRISMA is an abbreviation of Prevention and Recovery Information System for Monitoring and Analysis, and is often applied for root cause analyses in healthcare. Schaaf & Habraken defined four root causes: technical, organizational, human behavior and patient. We adapted these categories prior to the start of the data collection based on our experience with patient record reviewing in several Dutch hospitals [91]. We sub-divided the root causes “organization, human and patient” in organization (integrated care), organization (hospital department level), human (care provider), human (informal caregiver), patient (self-management) and patient (disease). In addition, we provided examples of causes for the settings: hospital care, ambulatory care and home (care). Hospital care was defined as the complete care process from admission until discharge, including outpatient visits after discharge. Ambulatory care was defined as medical care provided in an outpatient setting. Care at home was defined as informal care provided at home after discharge.

Two researchers (RS, MdB, CB, FK independently) allocated each cause to the root causes mentioned above. A third senior researcher performed a double check on the final cause classification. Disagreements between researchers were resolved during meetings (RS, CB, FK, MdB) until consensus was reached.

A few studies combined multiple types of causes within one single cause. We separated these different causes in order to allocate each of them to the most appropriate root cause. This resulted in a higher number of causes than there were originally present in the study. Furthermore, we were not able to allocate all causes to one root cause because some causes lacked a clear description of the context. We classified these causes as *Unclassifiable*. Specific categories of causes such as planned readmissions or unrelated readmissions were not considered for further analysis as these were not root causes for a readmission. After allocating all the causes, a final cause classification framework was constructed. Data collection and analyses were documented in Microsoft Excel 2016.

## Results

After applying the detailed inclusion and exclusion criteria 32 studies were excluded for this review, leaving 45 studies for further analysis (see Additional file 2b). The 32 studies were excluded because of at least one of the following reasons: no original data [16–18], no primary focus on preventable readmissions [15, 19–22], no clear description of methods and results (e.g. no distinction in preventability or type of admission [23–44] and no description of causes [45]. An overview of the excluded studies is shown in Additional file 4.

### Study design and characteristics

A summary of the study characteristics is shown in Table 1. The majority of the studies (64.4%) was conducted in the US and all studies were performed in a hospital setting (75.6% monocenter studies). Eight studies (17.8%) examined all-cause readmissions, meaning that these studies included readmissions from all departments, irrespective of whether this department was similar to that of the previous admission [46–53]. The majority of the studies included readmissions of a specific specialty (31.1%) [54–67] such as readmissions to a pediatric department. Twenty-seven percent of the studies examined readmissions of a specific group to a specific specialty (26.6%) [14, 68–78] such as patients with diabetes readmitted to internal medicine. Twenty-four percent of the studies included readmissions of a specific group (24.4%), such as elderly [2, 3, 13, 79–86]. Other studies included readmissions occurring to any department for a specific group (e.g. elderly to internal medicine).

**Table 1:** Descriptive characteristics of included studies that reported causes* (*n* = 45)

	Auteur	Publication year	Country	Study design	Setting	Disease group/department of index admission	Duration	Additional data source	Questions/topics available
1	Agrawal	2015	USA	retrospective	monocenter	Decompensated cirrhosis	30 days	NA	NA
2	Auerbach	2016	USA	cross-sectional	multicenter	General medicine	30 days	Interviews with patients; surveys of patients’ physicians	Topics
3	Balla	2008	Israel	cross-sectional	monocenter	Medicine	30 days	Interviews with patients	Topics
4	Bianco	2012	Italy	cross-sectional	monocenter	Medical or surgical illness	30 days	Interviews with patients	NR
5	Burke	2016	USA	retrospective	monocenter	Internal medicine	7 days and 30 days	Interviews with patients (pilot)	NR
6	Cakir	2010	USA	retrospective	monocenter	Not specified	30 days	NA	NA
7	Cooksley	2015	England	retrospective	monocenter	Oncology	30 days	NA	NA
8	Clarke	1990	England	retrospective	multicenter	General medicine/surgery, geriatrics	< 28 days/0–6 days and 21–27 days	NA	NA
9	Dawes	2014	USA	retrospective	monocenter	General surgery	30 days	NA	
10	Feigenbaum	2012	USA	cross-sectional	multicenter	Not specified	30 days	Interview with patients, providers and family	Questions and topics
11	Frankl	1991	USA	prospective	monocenter	Internal medicine	30 days	NA	NA
12	Glass	2013	USA	retrospective	multicenter	Patients undergoing pancreatectomy	30 days	NA	NA
13	Greenberg	2016	USA	retrospective	monocenter	Patient with a aneurysmal subarachnoid haemorrhage	30 day	NA	
14	Haines-Wood	2016	USA	retrospective	monocenter	All departments	15 days	NA	NA
15	Halfon	2002	Switserland	prospective	monocenter	All departments	30 days and 365 days	NA	NA
16	Harhay	2013	USA	retrospective	monocenter	Kidney transplantation	30 days	NA	NA
17	Jiminez-Puente	2004	Spain	cross-sectional	monocenter	All departments	6 months	NA	NA
18	Jonas	2016	USA	retrospective	monocenter	Paediatrics	15 days	NA	NA
19	Kelly	2015	UK	retrospective	monocenter	Learning disability	30 days	NA	NA
20	Maurer	2004	Switserland	prospective	monocenter	Internal medicine	90 days	NA	NA
21	Meisenberg	2016	USA	retrospective	monocenter	Oncology	30 days	NA	NA
22	Miles	1999	USA	retrospective	monocenter	All departments	28 days	NA	NA
23	Mittal	2016	USA	retrospective	multicenter	Acute ischemic stroke	30 days	NA	NA
24	Nahab	2012	USA	retrospective	monocenter	Stroke and cerebrovascular disease	30 days	NA	NA
25	Nijhawan	2015	USA	retrospective	monocenter	HIV patients	30 days	NA	NA
26	Oddone	1996	USA	prospective	multicenter	General medicine	6 months	NA	NA
27	Pace	2014	Canada	prospective	multicenter	Medical wards	30 days	NA	NA
28	Phelan	2009	Ireland	retrospective	monocenter	Heart failure	365 days	NA	NA
29	Ryan	2014	USA	retrospective	monocenter	Heart failure	30 days	NA	NA
30	Saunders	2015	USA	retrospective	monocenter	Oncology	30 days	NA	NA
31	Shah	2013	UK	retrospective	monocenter	Neurosurgery	30 days	NA	NA
32	Shalchi	2009	UK	retrospective	monocenter	Acute medical assessment unit	14 days	NA	NA
33	Shimizu	2014	USA	cross-sectional	monocenter	Internal medicine	30 days	Interviews with patients	Topics
34	Stein	2016	USA	cross-sectional	monocenter	Internal medicine	30 days	Interviews with patients	Questions and topics
35	Sutherland	2016	UK	retrospective	monocenter	Colorectal Surgery	30 day	Interviews with patient/ caretaker and attending surgeon	Topics
36	Tejedor-Sojo	2015	USA	retrospective	monocenter	Paediatrics	30 days	NA	NA
37	Toomey	2016	USA	cross-sectional	monocenter	Paediatrics	30 day	Interviews with parents/guardians, patients, inpatient clinicians and primary care providers	NR
38	Vachon	2012	USA	retrospective	monocenter	Trauma patients	30 days		
39	Van Walraven	2011	Canada	prospective	multicenter	Medicine and surgery	6 months	Interview with patients	Topics
40	Vinson	1990	USA	prospective	monocenter	Congestive heart failure	90 days	Interview with patient or family	NR
41	Wallace	2015	USA	retrospective	monocenter	Paediatrics	30 days	NA	NA
42	Wasfy	2014	USA	retrospective	multicenter	Patients undergoing percutaneous coronary intervention	30 days	NA	NA
43	Weinberg	2016	USA	retrospective	monocenter	Total hip arthroplasty	30 or 90 days	NA	NA
44	Williams	1988	UK	cross-sectional	monocenter	Geriatrics/all departments	28 days	Interviews with patient/carer/hospital ward sister and GP	NR
45	Yam	2010	China	retrospective	multicenter	Medicine	30 days	NA	NA

*= Including synonyms for causes such as (contributing) factors which were considered as a cause for readmissions

Eight studies referred to an existing root-cause assessment tool with predefined causes such as STAAR, PRISMA or REDcap tool [2, 53, 65, 69, 76, 81, 84, 85]. Multiple studies referred to causes used in previous publications [3, 50, 55, 57, 59–61, 64, 66, 67, 71, 74, 75, 79]. In particular, the studies of Clarke [59], Goldfield [87], Jiminez-Puente [49] and Oddone [74] were frequently referred to.

### Cause classification

The frequencies of each root cause are listed in Additional file 5. A total of 381 causes were found of which 275 were reported as preventable and 44 as unpreventable (see Additional file 5). Twenty-six causes were reported as both preventable and unpreventable in a single study, and these causes were coded as both. Examples of causes that were considered differently among studies are listed in Table 2. Other causes that were not explicitly defined as preventable or unpreventable (*n* = 36) were coded as neutral. The final cause classification framework is listed in Table 3.

**Table 2:** Causes that were reported as both preventable and unpreventable

Cause	Example - preventable	Example - unpreventable
Complication	Foreseen complication [61, 79]; complication of surgical procedures [48, 49, 81]; post-procedure complications and complications related to medication use [63]	Complications related to neurological impairment and immobility [70]; complications occurring in spite of best practices being followed, complications due to progression of natural history of certain chronic neurosurgical disease [63]
Adherence	Reasons probably within control of hospital services (may include compliance [59]; patient compliance [51]; non-compliance with prescribed medication [82]; improved medication compliance [14]; medication non-compliance [77]; caretaker related - Noncompliance with discharge medications [86]; Noncompliance that could have been prevented by care givers [78]; patient compliance problems [67]; fluid related - nomcompliance diet and medication and hepatic encephalopathy - noncompliance to medication [54]	Reasons probably beyond control of hospital services (may include compliance [61]
Disease progression	Disease progression [64, 78]; Care during index stay - Unrecognized worsening condition [47];	Unavoidable recurrence or progression of disease [49]; Disease progression [64]; disease related - Unforeseen worsening of disease process [86]

**Table 3:** Final cause classification framework

Root cause	Definition	Includes	References
Technical	Defect materials, poor design of material or inaccessible material.		
Organization – integrated care	Failures at integrated care level such as coordination and communication problems.	Coordination; Admissions for tests, procedures, or treatments that could have been performed in the previous admission; quality management (assurance and control); Responsibilities; Better use of community services, inappropriate discharge setting or appropriate discharge setting not available, care could have been provided in; primary care setting; Problems with healthcare transitions; social readmission; Suboptimal primary care case management, lack of home health/home physical therapy visit, earlier PCP follow-up necessary	[13, 48, 51, 53, 55, 57–61, 64, 65, 67, 72, 74, 79, 80, 82]
Organization - department level care	Failures related to inadequate organization of care for a single patient. These failures may be related to clinical processes such as diagnostics, medication, surgical procedure, surgical complications.	Surgical and non-surgical, disease-specific complications, general complications (nosocomial infection, wound complication, dehydratation, bleeding). Suboptimal drug treatment, error in drug prescription, overdosing, suboptimal medication reconciliation. Proper diagnostics not performed or not timely performed. Inadequate pain control, closer management/monitoring of comorbid disease, delay in palliative care consultation. Lack of discharge planning, early discharge. Patient education. Timely outpatient clinic visit scheduled	[3, 13, 14, 46–86]
Human - care provider	Failures resulting from shortcomings in skills and knowledge of the care provider.	Decision to admit patient, disregarding diagnostic results or concerns from collegeaues, wishful thinking, lack of experience to make a proper decision. Inadequate (clinical) skills and knowledge; or lack of experience. Patient not sufficiently monitored by care provider. Neglegence, fault	[2, 67, 68, 72, 74, 84, 86]
Human - informal caregiver	Inadequate support from informal caregiver.	Inadequate social support, wishful thinking	[13, 71, 77, 84–86]
Patient - selfmanagement	Incorrect behavior of the patient that may include incompliance, abuse of medication, non-adherence.)	Not showing up for follow-up care, non-compliance with medication or diet; substance abuse; patient coping, wishful thinking, lack of knowledge, patient preference (leaving against medical advice)	[13, 14, 47, 48, 51, 53–55, 58, 59, 61, 64, 67, 68, 71, 74, 75, 77, 79, 81, 82]
Patient - disease	Unexpected complications related to the patient’s condition (disease progression, comorbidity, severity of illness).	Unavoidable complication; unavoidable disease progression; chronic or relapsing disorder.	[3, 13, 48–50, 53, 58, 59, 61, 64, 66, 67, 70, 72, 79–82, 86]
Unclassifiable	Causes which cannot be allocated to one of the other themes because the cause has an ambiguous meaning		[13, 14, 47, 59, 65, 69, 71, 74, 75, 78, 82, 85]

#### Technical

None of the studies reported technical failures as a cause for preventable readmissions.

#### Organization – integrated care

Eighteen studies reported causes related to the organization of integrated care [13, 48, 51, 53, 55, 57–61, 64, 65, 67, 72, 74, 79, 80, 82]. Fifteen studies considered these causes as preventable [48, 51, 57–61, 64, 65, 67, 72, 74, 79, 80, 82]. Social readmissions, unavailability of outpatient care and system failures were frequently considered as preventable. One study reported that hospitals were partly accountable for social readmissions [48], while other studies reported that social readmissions were related to inadequate care by the caretaker/spouse [84, 86]. Two studies stated that causes related to the organization of integrated care were seen as unpreventable [64, 72]. For example, Jonas described the lack of alternative resources as an unpreventable cause [72].

#### Organization – hospital department level

The majority of the studies (*n* = 44; 97.8%) described causes related to the organization of care at hospital department level [3, 13, 14, 46–86]. Many causes were related to the coordination and planning of care. Feigenbaum et al. (2012) reported factors contributing to potentially preventable readmissions such as “suboptimal coordination of care during index stay” and “inadequate arrangement of supplies during discharge process” [47]. In addition, Greenberg et al. (2016) reported that a readmission could be prevented if the disposition of planning and the coordination of care would have been adequate. Forty-two studies described these causes as preventable [3, 14, 46–52, 54–86]. Readmissions related to complications caused by the care provided during the previous admission were often reported as a preventable cause [2, 3, 11, 47–49, 52, 54, 59, 61–64, 66, 72, 76, 78, 79, 81, 84, 85].

Nine studies also described unpreventable causes related to the organization of patient care [50, 61, 63, 64, 69, 70, 72, 75, 80]. Some studies reported complications related to a specific patient population [70, 72, 76]. For example, the study of Vachon et al. (2012) focused on trauma patients and included the following complications: wounds, abdominal, pulmonary, thromboembolic, central nervous system, hematoma and other [76].

#### Human – care provider

Seven studies reported causes related to the care provider [2, 67, 68, 72, 74, 84, 86], such as inadequate decision making, inadequate (clinical) skills and knowledge, or lack of experience. In all cases, these causes were considered to be preventable. Examples of these causes include: ‘Failure resulting from faulty task planning or performance’ and ‘Failure of an individual to apply their knowledge to a new clinical situation’ [2].

#### Human – informal caregiver

Six studies described causes related to the informal caregiver [13, 71, 77, 84–86]. All these causes were seen as preventable. For example, Harhay and Vinson described inadequate support at home as a cause for preventable readmissions.

#### Patient – self-management

Twenty-one studies included causes related to self-management of the patient [13, 14, 47, 48, 51, 53–55, 58, 59, 61, 64, 67, 68, 71, 74, 75, 77, 79, 81, 82]. Ten studies described causes related to self-management as preventable [14, 47, 48, 51, 54, 55, 58, 64, 67, 68, 71, 74, 75, 77, 82]. Non-adherence was also frequently reported as a preventable cause [14, 51, 54, 59, 61, 67, 71, 74, 77, 82]. For example, Yam described adherence problems and patient coping as causes for preventable readmissions. In six studies, causes related to self-management were seen as unpreventable [58, 59, 61, 64, 79, 81]. For example, Burke considered causes such as adherence to discharge plan, refusal of discharge plan and substance use as unpreventable.

#### Patient – disease

Many studies reported causes related to the patient’s disease [3, 13, 48–50, 53, 58, 59, 61, 64, 66, 67, 70, 72, 79–82, 86]. Ten studies considered disease related causes as potentially preventable causes. Clarke reported ‘recurrence or continuation of disorder leading to first admissions’ as a preventable disease-related cause [59]. Disease progression (*n* = 16) was frequently reported as unpreventable. Examples of the disease-related causes were; chronic or relapsing disorder [59, 61, 79], unforeseen worsening of disease progress and closely related conditions [49, 58, 72], acute myocardial ischemia and poorly controlled arrhythmias [82].

#### Unclassifiable

Twelve studies reported some causes that could not be classified into root causes [13, 14, 47, 59, 65, 69, 71, 74, 75, 78, 82, 85]. Causes such as problems with services can apply to institutions, departments or individuals. For example, patient assessment was mentioned as a cause for readmission, but it was not specified who performed the patient assessment or what was exactly meant with it.

## Discussion

### Purpose of study and significant conclusions

The main objective of this systematic review was to summarize the range of causes described for unplanned readmissions and to present a cause classification framework for causes of readmissions. The findings of this review show that many studies primarily focus on causes related to organization of care at the department level. The causes were mainly evaluated from a hospital perspective. None reported technical failures as one of the causes of (preventable) readmissions. We succeeded in classifying the majority of the causes according to the adapted PRISMA scheme and to distinguish between levels of care. We were not able to allocate causes that were ambiguous.

### Key results compared to other studies

The results of this review indicate that the causes of readmissions depend on the context of the study, e.g. the inclusion of department/healthcare organization, case-mix, assessor of preventability, and definition of preventability. All studies were conducted in the hospital and several studies included causes beyond the setting of the hospital. The latter studies often identified other causes for readmissions in addition to hospital care (e.g. social support [13, 71, 77, 84–86]). This finding is in line with the existing literature [6, 11, 88–90]. Zhou et al. (2017) reviewed studies which used prediction models for (preventable) unplanned readmissions. They identified that health insurance and overall prognosis should be considered as contributors in addition to the clinical factors which are normally included in the statistical models. We also found that factors such as self-management, social support, and type of healthcare organization can play a role. Other factors beyond the scope of the hospital should be assessed in more detail to gain a better insight into the complexity behind unplanned readmission and to understand where interventions can have the most impact. In addition, gaining more understanding on these factors is necessary in order to optimize the applicability of unplanned readmissions as a quality indicator of integrated care [11, 88]. Decreasing unplanned readmissions needs a holistic approach, because health care is a complex dynamic system in which patients, professionals, health care organization are acting within the regulations of their national health care system.

Furthermore, this review highlights that studies differ in which causes are considered to be preventable. In particular, complications, adherence and disease progression were seen as both preventable and unpreventable. For example, ‘foreseen complications’ were seen as preventable [61, 79]. Complications of surgical procedures were also reported as preventable in many studies [48, 49, 81]. Greenberg et al. reported complications related to neurological impairment and immobility as an unpreventable cause [70]. Shah et al. considered complications as preventable and unpreventable. They defined the following complications: complications occurring in spite of best practices being followed, complications due to progression of natural history of certain chronic neurosurgical disease, post-procedure complications and complications related to medication use. The first two were reported as unpreventable, and the latter two as preventable [63]. These findings highlight that preventability can differ depending on many contextual factors and what the authors of studies regard as adequate care.

The proposed classification allows researchers and policymakers to further consider the complex nature of readmissions. Differences in cause classifications can be problematic for fair comparison of studies. Therefore, it is essential that studies describe the context of the research setting/research population and elaborate on what they regard as preventable.

In addition, this review indicates that the assessment of readmissions is mainly based on data that is inputted in the hospital information system. Some studies included interviews and were thereby able to capture the patients’ experience. However, the transcripts were not available and therefore not included in this review. Furthermore, studies indicated that interviews with patients and/or caregivers can provide additional information on factors beyond the hospital setting which cannot be found in medical records [85]. For instance, Sutherland et al. (2016) found that the combination of patient interviews and chart review revealed additional gaps in care [75]. In addition, Toomey et al. (2014) found in 31.2% of cases that interviews with patients and primary care providers provided new information. These findings support other studies stating that increased involvement of patients and other stakeholders are crucial for assessing the cause of the readmission and may contribute to the prevention of readmissions and to better quality of care [89].

### Strengths and limitations

This review provides insight into the range of causes reported in unplanned readmission studies. The insights gained from this review may help others in conducting readmission studies. However, this review has some limitations. Firstly, the studies included were based on a set of articles which were collected for a previous review [10]. As a consequence, we may have missed studies which also examined the causes of unplanned readmissions, but did not provide information on the rate of preventable readmissions. This might have limited the number of causes reported in this study and consequently the number of root causes/categories in our cause classification framework. However, we performed a cross reference checking in Scopus and Web-of-Science and did not find additional studies. Secondly, we allocated a single cause to only one root cause. Thus, causes which consisted of multiple sub-causes were disentangled into separate causes. This might have influenced the frequency of the themes and subthemes that were found. Thirdly, studies which did not explicitly specify the context of a specific cause (ambiguous causes) were considered “*Unclassifiable*”. As a result, the number of causes related to other root causes may be underestimated. Lastly, all phases were either consensus based-driven and/or performed by at least two independent data extractors. However, using this procedure can not preclude that some amount of interpretation bias occurs during data collection, synthesis and interpretation.

### Recommendations for policy

Since 2016, unplanned readmissions have been used as a quality indicator for hospital care in the Netherlands. To increase the comparability of causes between readmission studies, we advise that the use of a standardized cause classification framework should be stimulated. The framework proposed in this review provides a broad range of causes which are most frequently observed. Regarding hospital care at the department and institutional level this overview is rather complete, although technical causes are lacking and are becoming more relevant due to development of eHealth, mHealth and ICT supported long distance care. Causes at the patient and health care system level are still of limited detail. For example, more information on the patient experience can be collected using interviews. Insight into these causes is crucial for developing improvement opportunities.

In addition, the proposed framework may contribute to a transparent development of prediction models. Information on how data sources are used to calculate the readmission indicator are often lacking [11]. Current prediction models depend on a limited range of data documented in the hospital information system. Information on what happens after discharge is (often) not documented. Hence, policy makers should be careful in holding hospitals accountable for unplanned readmissions as long as it is unclear which percentage of these readmissions is truly preventable by preventive actions of the hospital itself. Unplanned readmission should be viewed as adverse events occurring in a complex dynamic system, in which patients, professionals and organizations all play a crucial role, regulated by the healthcare system.

### Recommendations for research

This review indicates that the causes of unplanned readmissions which are examined by the studies differ between studies and are often limited to causes focused on hospital care. As a result, the multifactorial nature of readmissions is not well understood and certain contributing causes might be overlooked. This limits the potential impact of (patient-centered) interventions to prevent readmissions [88]. To improve our understanding of readmissions, all actors involved in the patient journey should be considered. The suggested framework may provide direction on which causes to include in a readmission study and prediction models. In the future, we hope that with the use of prediction models, high-risk patients can be more easily identified and targeted for alternative management [90]. In addition, future research should take into account the interdependency of causes. For instance, when a patient gets readmitted due to an incorrect assessment by a physician at discharge, this readmission might be the result of how readmissions are handled at a department/organization. Physicians might not be effectively trained to ask the patient if they are ready to be discharged. Lack of skills can thus also be considered a result of suboptimal organization of care (organization department level care). Failure on organization level (e.g. missed opportunity to train physicians) or care-provider level (lack of skills) are important to consider. In addition, it is also possible that failure on one level might be mitigated by other levels. Each level must be considered in the study of readmissions. Multiple causes of readmissions - in and beyond the hospital - play a role. All these causes should be assessed to capture the complex nature of readmissions and should be considered interdependently.

## Conclusion

In conclusion, we aimed to: [1] evaluate the range of causes for unplanned readmissions, and [2] present a cause classification framework for causes related to readmissions. The results show that the causes of readmissions used differ considerably among the studies. The current use of different causes limits the opportunity to compare studies and to learn from unplanned readmissions. A shared vision on unplanned readmissions is necessary to improve the uniformity and transparency on the causes of readmissions. This can be achieved by ordering all causes in the new cause classification framework based on the PRISMA cause classification. The new cause classification framework may contribute to the standardization of designing and conducting readmission studies, and the comparability of readmission studies. The findings of this review may help us to understand the complex nature of readmissions and emphasize the importance of using a broad range of causes that may occur on the patient’s journey when examining unplanned readmissions. As described by the studies, unplanned readmissions can be caused by many factors at all levels of the health care system throughout all the phases of the patient journey.

## Additional files

Additional file 1: Pubmed search strategy. (DOCX 14 kb)

Additional file 2: PRISMA flowchart reviews. (DOCX 66 kb)

Additional file 3: Flowchart Inclusion and exclusion criteria. (DOCX 25 kb)

Additional file 4: Excluded studies and reason for exclusion for review. (XLSX 23 kb)

Additional file 5: Root cause and causes as reported in articles. (XLSX 29 kb)

## Data Availability

The datasets supporting the conclusions of this article are included within the article or provided in the additional files.

## Abbreviations

PRISMA (flowcharts): Preferred Reporting Items for Systematic Reviews and Meta-Analyses
PRISMA (root cause analysis tool): Prevention and Recovery Information System for Monitoring and Analysis
REDcap tool: Research Electronic Database Capture
STAAR: State Action on Avoidable Rehospitalizations
