# Correction to: Towards a patient journey perspective on causes of unplanned readmissions using a classification framework: results of a systematic review with narrative synthesis

**DOI:** 10.1186/s12874-019-0851-4

**Published:** 2019-11-27

**Authors:** R. G. Singotani, F. Karapinar, C. Brouwers, C. Wagner, M. C. de Bruijne

**Affiliations:** 1 Department of Public and Occupational Health, Amsterdam Public Health research institute, Amsterdam UMC, Vrije Universiteit Amsterdam, Van der Boechorststraat 7, NL-1081 BT Amsterdam, The Netherlands; 2 Department of clinical pharmacy, Onze Lieve Vrouwe Gasthuis (OLVG), location West, Jan Tooropstraat 164, 1061 AE Amsterdam, The Netherlands; 3 Netherlands institute for Health Services research, Otterstraat 118-124, 3513 CR Utrecht, The Netherlands

**Correction to: BMC Med Res Methodol**

**https://doi.org/10.1186/s12874-019-0822-9**

Due to an error introduced during copyediting of this article [1], following corrections need to be made:

1. Reference 15/91 (*Zegers, M., De Bruijne, M. C., Wagner, C., Hoonhout, L. H. F., Waaijman, R., Smits, M., & Groenewegen, P. P. (2009). Adverse events and potentially preventable deaths in Dutch hospitals: results of a retrospective patient recordreview study. BMJ Quality & Safety, 18(4), 297-302*) is reported twice in the reference list. It should be #91.
2. Reference 15 should be: *El-Jawahri A, Keenan T, Abel GA, Steensma DP, LeBlanc TW, Chen Y-B, et al. Potentially avoidable hospital admissions in older patients with acute myeloid leukaemia in the USA: a retrospective analysis. The Lancet Haematology. 2016;3(6):e276–e83*
3. The reference to additional file 4A and 4B is incorrect (see Authors’ contribution: *Authors’ contributions Two researchers (RS and CB) independently screened the set of articles included in the primary selection for eligibility for this review (according to the inclusion and exclusion criteria (see: Additional file 4A and B)*. It should be additional file 2A and 2B instead of 4A and 4B.
4. Table 1 (Excluded studies and reason for exclusion for review) should be added to the Additional file 4. This amendment will change the numbers of the tables and files in the manuscript. It changes as follows:

The original article has been corrected.

**Table:** 

Sub-paragraph and page	Current sentence	Change in text:
Results, p3	An overview of the excluded studies is shown in Table 1	An overview of the excluded studies is shown in Additional file 4
		Add to [new] Additional file 4 – Footnote (see file)
Results, p5	Title of table: Table 1 Excluded studies and reason for exclusion for review	Additional file 4: Excluded studies and reason for exclusion for review
Results, p3	A summary of the study characteristics is shown in Table 2.	A summary of the study characteristics is shown in Table 1.
Results, p6	Table 2 Descriptive characteristics of included studies that reported causes* (*n* = 45)	Table 1 Descriptive characteristics of included studies that reported causes* (*n* = 45)
Results, p7	Table 2 Descriptive characteristics of included studies that reported causes* (*n* = 45) (Continued)	Table 1 Descriptive characteristics of included studies that reported causes* (*n* = 45) (Continued)
Results, cause classification, p4	Examples of causes that were considered differently among studies are listed in Table 3.	Examples of causes that were considered differently among studies are listed in Table 2.
Results, cause classification, p4	Table 3 Causes that were reported as both preventable and unpreventable	Table 2 Causes that were reported as both preventable and unpreventable
Results, cause classification, p4	The final cause classification framework is listed in Table 4.	The final cause classification framework is listed in Table 3
P9	Table 4 Final cause classification framework	Table 3 Final cause classification framework
Additional files list, p10		Additional file 4: Excluded studies and reason for exclusion for review
Results, cause classification, p4	The frequencies of each root cause are listed in Additional file 4. A total of 381 causes were found of which 275 were reported as preventable and 44 as unpreventable (see Additional file 4).	The frequencies of each root cause are listed in Additional file 5. A total of 381 causes were found of which 275 were reported as preventable and 44 as unpreventable (see Additional file 5).
Additional files list, p10	Additional file 4: Root cause and causes as reported in article	Additional file 5: Root cause and causes as reported in article

## Supplementary information

**Additional file 4.** Excluded studies and reason for exclusion for review.
