# Supplementary material for: Towards a patient journey perspective on causes of unplanned readmissions using a classification framework: results of a systematic review with narrative synthesis
Source: BMC Med Res Methodol. 2019 Oct 4;19:189. doi: 10.1186/s12874-019-0822-9 (PMC6778387; doi:10.1186/s12874-019-0822-9)
Supplement: Supplementary file 2 — PRISMA flowchart reviews. (DOCX 66 kb) [file 12874_2019_822_MOESM2_ESM.docx]

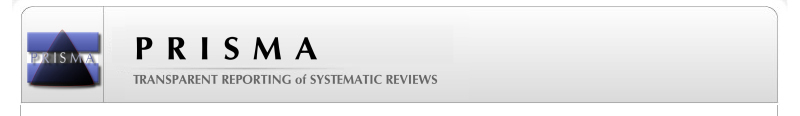
**Additional file 2: Fig. S1. PRISMA 2009 Flow Diagram – Previous review (2017)**

**Screening**

**Included**

**Eligibility**

**Identification**

Records identified through database searching *(PubMed, Embase, Scopus, Web of Science)*

(n = 4659)

Additional records identified through other sources
(n = 18)

Records after duplicates removed
(n = 2504)

Records screened
(n = 2155)

Records excluded
(n = 2052)

Full-text articles assessed for eligibility
(n = 103)

Full-text articles excluded, with reasons
(n = 26)

Studies included in qualitative synthesis
(n = 77)

Studies included in quantitative synthesis
(n = 49)


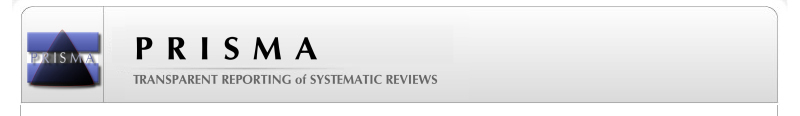
**Additional file 2: Fig. S2. PRISMA 2009 Flow Diagram – Current review (2018)**

**Screening**

**Included**

**Eligibility**

**Identification**

Records identified through database searching *(PubMed, Embase, Scopus, Web of Science)*

(n = 4659)

Additional records identified through other sources
(n = 18)

Records after duplicates removed
(n = 2504)

Records screened
(n = 2155)

Records excluded
(n = 2052)

Full-text articles assessed for eligibility
(n = 103)

Full-text articles excluded, with reasons
(n = 26)

Additional full-text assessment for eligibility
(n = 77)

Studies included in data synthesis
(n = 45)

Full-text articles excluded, with reasons
(n = 32)
