# Supplementary material for: Towards a patient journey perspective on causes of unplanned readmissions using a classification framework: results of a systematic review with narrative synthesis
Source: BMC Med Res Methodol. 2019 Oct 4;19:189. doi: 10.1186/s12874-019-0822-9 (PMC6778387; doi:10.1186/s12874-019-0822-9)
Supplement: Supplementary file 3 — Flowchart Inclusion and exclusion criteria. (DOCX 25 kb) [file 12874_2019_822_MOESM3_ESM.docx]

**Additional file 3: Flowchart Inclusion and exclusion criteria**

**Inclusion criteria:**

1. Is the paper written in English?
2. Is the study based on original data?
3. Is the primary objective of the paper focused on (unplanned) hospital readmissions?
4. Is the preventability of (unplanned) readmissions assessed?
5. Is the assessment of preventability based on medical chart review or, in case of survey/ interview etc., based on individual patient data?
6. Were the causes (≥3) of potentially preventable readmissions discussed in the method and or result section?
