# Supplementary material for: Towards a patient journey perspective on causes of unplanned readmissions using a classification framework: results of a systematic review with narrative synthesis
Source: BMC Med Res Methodol. 2019 Oct 4;19:189. doi: 10.1186/s12874-019-0822-9 (PMC6778387; doi:10.1186/s12874-019-0822-9)
Supplement: Supplementary file 4 — Excluded studies and reason for exclusion for review. (XLSX 23 kb) [file 12874_2019_822_MOESM4_ESM.xlsx]

**Additional file 4: Excluded studies and reason for exclusion for review^[[1]](#endnote-1)^**

|  | **Author** | **Publication year** | **Reason for exclusion** |
| --- | --- | --- | --- |
| 1 | Allen-liles | 2015 | 6 |
| 2 | Amin | 2016 | 6 |
| 3 | Bell | 2015 | 6 |
| 4 | Borzecki | 2015 | 3 |
| 5 | Brooks | 2014 | 3 |
| 6 | El-Jawahari | 2016 | 3 |
| 7 | Epstein | 2015 | 6 |
| 8 | Experton | 1999 | 6 |
| 9 | Fluitman | 2016 | 6 |
| 10 | Gautam | 1996 | 6 |
| 11 | Gilotra | 2016 | 6 |
| 12 | Graham | 1983 | 6 |
| 13 | Hain | 2013 | 6 |
| 14 | Halfon | 2006 | 2 |
| 15 | Hauviller | 2016 | 6 |
| 16 | Hechenbleikner | 2013 | 6 |
| 17 | Herzig | 2015 | 2 |
| 18 | Jackson | 2014 | 2 |
| 19 | Kirk | 2006 | 6 |
| 20 | Koekkoek | 2011 | 7 |
| 21 | Lee | 2017 | 6 |
| 22 | Levy | 2000 | 6 |
| 23 | Ludke | 1990 | 6 |
| 24 | McIntyre | 2016 | 6 |
| 25 | Njeim | 2012 | 6 |
| 26 | Patel | 2016 | 6 |
| 27 | Ruiz | 2008 | 3 |
| 28 | Sutton | 2002 | 6 |
| 29 | Trickey | 2016 | 6 |
| 30 | Volk | 2012 | 6 |
| 31 | Witherington | 2008 | 3 |
| 32 | Yap | 2016 | 6 |

1. Inclusion criteria (See also: Additional file 3)

   1. Is the paper written in English?

   2. Is the study based on original data?

   3. Is the primary objective of the paper focused on (unplanned) hospital readmissions?

   4. Is the preventability of (unplanned) readmissions assessed?

   5. Is the assessment of preventability based on medical chart review?

   6. Were the causes (≥3) of potentially preventable readmissions discussed in the method and or

   result section? [↑](#endnote-ref-1)
